# Unearthing soil-plant-microbiota crosstalk: Looking back to move forward

**DOI:** 10.3389/fpls.2022.1082752

**Published:** 2023-01-24

**Authors:** Marco Giovannetti, Alessandra Salvioli di Fossalunga, Ioannis A. Stringlis, Silvia Proietti, Valentina Fiorilli

**Affiliations:** ^1^ Department of Biology, University of Padova, Padova, Italy; ^2^ Department of Life Sciences and Systems Biology, University of Turin, Turin, Italy; ^3^ Plant - Microbe Interactions, Department of Biology, Science4Life, Utrecht University, Utrecht, Netherlands; ^4^ Department of Ecological and Biological Sciences, University of Tuscia, Viterbo, Italy

**Keywords:** soil quality, soil-microbe-plant system, microbiome, plant health, soil database, soil indicators

## Abstract

The soil is vital for life on Earth and its biodiversity. However, being a non-renewable and threatened resource, preserving soil quality is crucial to maintain a range of ecosystem services critical to ecological balances, food production and human health. In an agricultural context, soil quality is often perceived as the ability to support field production, and thus soil quality and fertility are strictly interconnected. The concept of, as well as the ways to assess, soil fertility has undergone big changes over the years. Crop performance has been historically used as an indicator for soil quality and fertility. Then, analysis of a range of physico-chemical parameters has been used to routinely assess soil quality. Today it is becoming evident that soil quality must be evaluated by combining parameters that refer both to the physico-chemical and the biological levels. However, it can be challenging to find adequate indexes for evaluating soil quality that are both predictive and easy to measure *in situ*. An ideal soil quality assessment method should be flexible, sensitive enough to detect changes in soil functions, management and climate, and should allow comparability among sites. In this review, we discuss the current *status* of soil quality indicators and existing databases of harmonized, open-access topsoil data. We also explore the connections between soil biotic and abiotic features and crop performance in an agricultural context. Finally, based on current knowledge and technical advancements, we argue that the use of plant health traits represents a powerful way to assess soil physico-chemical and biological properties. These plant health parameters can serve as *proxies* for different soil features that characterize soil quality both at the physico-chemical and at the microbiological level, including soil quality, fertility and composition of soil microbial communities.

## Introduction

Life on Earth and its biodiversity depend on the soil (Guerra et al., 2021). However, the soil is a non-renewable and threatened resource extremely vulnerable to climate change and to intensive agroecosystem management (Lehmann et al., 2020). Therefore, it is urgent to maintain and improve soil biodiversity and fertility considering that soil quality is critical to ecological balances, food production and human health. Indeed, soil underpins a range of ecosystem services because of its pivotal role in regulating water flow and storage, gaseous exchanges, nutrient dynamics, food security and crop productivity (Brevick et al., 2018). Soil is composed of an extremely complex matrix of organic and inorganic materials. Different physico-chemical and biological parameters influence this matrix and determine soil properties and functions (Kekane et al., 2015). Nevertheless, one of the hardest challenges of soil science is deciphering soil’s complexity to assess its quality and measuring soil parameters can require advanced equipment depending on the focus. In this review, we present how the focus of soil science is gradually shifting from physico-chemical analysis toward biological components (*i.e.* soil microbial community and activity) and how we envision that overall plant health, and not just crop productivity, could represent a valuable readout for evaluating soil quality. We are conscious that the plant has been used as a primordial indicator to infer soil properties. Here we propose new arguments for using plant health as a reliable *proxy* of soil properties, as supported by the current scientific framework.

## Current “*status quo*” of soil quality indicators

Defining soil-related concepts is difficult because soil is complex and heterogeneous. In this review, we mainly focus on the soil quality concept as defined in **Table 1**, which emphasizes the services that soil provides to living organisms. In the last three decades, several authors have highlighted the importance of using indicators of a different nature to achieve a clear understanding of soil quality and as valuable assets for ecosystem monitoring and assessment (Muñoz-Rojas, 2018). Due to the multi-dimensional nature of soil quality, using multiple indicators is generally preferred. An assessment approach based on a set of indicators should be considered safer than relying on a single indicator. A soil quality indicator should be i) relevant ii) sensitive iii) practical, user-friendly and cheap, and iv) informative for management (Rinot et al., 2019). Soil quality indicators are traditionally divided into physical, chemical and biological, most of them being very well connected with each other (Cardoso et al., 2013; Muñoz-Rojas, 2018; Rakshit et al., 2020).

**Table 1:** Soil-related concepts and definitions (according to Bünemann et al., 2018; Lehmann et al., 2020).

Soil **fertility** is the oldest and narrowest concept and it refers to the ability of soil to support the growth of plants and to sustain the crop yield for human use. Fertile soil is important for agriculture, as it enables crops to grow healthy and strong, providing an abundant and nutritious food, fibre or fuel supply.
Soil **quality** is a measure of the soil ability to function effectively in support of plant growth and other ecosystem services. It is usually considered as “the capacity of a soil to function within ecosystem and land-use boundaries to sustain biological productivity, maintain environmental quality, and promote plant and animal health”, including humans (Doran and Parkin, 1994; Doran and Parkin, 1996).
Soil **health** has been used since the late 90s and it “captures the ecological attributes of the soil which have implications beyond its quality or capacity to produce a particular crop. These attributes are chiefly those associated with the soil biota; its biodiversity, its food web structure, its activity and the range of functions it performs” (Pankhurst et al., 1997). Soil health and quality are often used as synonyms, but soil health usually does not only refer to human-related ecosystem services.
Soil **security** refers to the idea that access to the services provided by soil ecosystems should be considered a fundamental human right and is often discussed in the context of policy making. It involves considering the cultural, financial, and legal aspects of soil management.

Guo (2021) proposed a further classification of indicators into 3 tiers: Tier 1 indicators, which have been widely accepted; Tier 2 indicators, which are regionally validated but need additional research for improved adoption; and Tier 3 indicators, which are promising to mirror soil quality, however extensive research is needed for improvements in measurement, interpretation, and use of them. Physico-chemical indicators are widely used and mostly reported in Tier 1. Physical soil parameters determine air and water movement; they are the most visible and their assessment does not require dedicated equipment. Most common physical indicators are bulk density, soil texture and structure, aggregate stability, porosity, plant available water and hydraulic properties (Horn et al., 1994). These indicators represent solid soil particles, which are often correlated with hydrogeological processes including erosion, aeration, runoff, infiltration rate, and water holding capacity and can have an impact on important soil characteristics. For instance, soil texture impacts the balance between water and gases (Oertel et al., 2016; Wang et al., 2021). Aggregate stability is greatly influenced by plants and soil microbial communities and impacts plant available water (Blankinship et al., 2016). On the other hand, available water capacity depends on soil texture. Chemical properties represent complex chemical reactions. Chemical parameters such as pH, organic and total carbon (C) and nitrogen (N), mineral nutrients such as phosphorus (P) and potassium (K), and cation exchange capacity are typically correlated with the potential to provide nutrients to plants and retain chemical compounds that are harmful to the biotic and abiotic environment (Schjoerring et al., 2019; Mohiuddin et al., 2022). Soil pH has a direct impact on physico-chemical and biological soil parameters. It can affect soil compactness (Matsumoto et al., 2018) and soil shear strength parameters (Ghobadi et al., 2014) through its action on soil water retention and is strongly related to nutrient availability and microbial activity (Parra et al., 2017; Lammel et al., 2018). Microbial activity is also affected by organic C, which impacts water holding capacity, stability of aggregates and storage of N (Smith et al., 2021). Nitrogen and P are the most important plant nutrients: they are both present in a variety of chemical forms, indicating their highly dynamic activity and their presence in the soil is largely depending on microbiological or physico-chemical dynamics, respectively (Shen et al., 2011; Fowler et al., 2013).

Since soils are major reservoirs of terrestrial C (Lal, 2008), soil organic carbon (SOC) is a key functional and manageable chemical indicator (Lal, 2016; Hueso-González et al., 2018). SOC is the major component of soil organic matter (SOM) and has critical roles in all soil processes. The annual rate of SOM losses can vary greatly, particularly in agricultural soils, and strictly depends on tillage methods, the type of plant/crop cover, drainage status of the soil and weather conditions. SOC is thus critical for climate change adaptation and mitigation strategies (Muñoz-Rojas, 2018). Biological indicators are reported in Tier 2 and Tier 3. They are represented by enzyme activity, microbial community composition, soil microbial biomass and activity, soil respiration rate, soil protein index, fatty acid methyl ester (FAME) profile, phospholipid fatty acid (PLFA) analysis, soil biodiversity, abundance of earthworms, presence of pathogens and parasites (Guo, 2021 and references therein).

To integrate indicators for soil quality into a soil management or conservation program, different approaches can be followed. One approach is to conduct soil surveys to gather data on the physical, chemical, and biological soil parameters. This can help to identify areas with poor or declining soil quality, and can provide a baseline for monitoring changes over time. A set of soil quality indicators that reflects the specific needs and goals of the program can also be developed. Indicators of soil quality can then be developed and used to guide the implementation of practices aimed at improving the soil. It’s important to regularly review and update these indicators, and to involve all relevant stakeholders in the process. Finally, recent advances in data analysis and machine learning techniques, such as network analysis and structural equation modeling, can be used to establish connections between soil quality indicators. This can provide powerful, interactive tools for soil assessment and management (Bünemann et al., 2018 and references therein).

Although their usage is becoming more and more frequently appreciated, the number and the type of biological indicators to construct a soil quality assessment system is still under debate (Paz-Ferreiro and Fu, 2013; Nunes et al., 2021; Guo, 2021). Surely, more scientific data are needed to further validate the soil quality relevance and practicality of these biological indicators. Here, we present evidence for microbiota being an emerging and valuable aspect of soil quality.

### Microbiota as determinants for soil quality

Microbial diversity and activity represent an aspect of soil quality. Bacteria, fungi, nematodes, protozoa and earthworms are part of the biological properties of the soil and their activity are interconnected with soil physical and chemical properties such as aeration, SOM or pH, and greatly contribute to C and nutrient cycling (Yang et al., 2018). The degradation of soils due to land-use change, erosion, compaction, and pesticide contamination has led to a decline in soil health and ecosystem services. To sustain these services and improve soil quality, it’s crucial to understand the role of microbial communities in soil health. Defining microbiota and microbiome, and using them as indicators of soil quality, is an important first step in this process. The plethora and diversity of microbes residing in the soil (bacteria, fungi, archaea, protists and algae) are collectively called soil microbiota, while microbiota together with their genomic, functional potential are collectively referred to as the microbiome (Pascale et al., 2020; Berg et al., 2020). High-throughput sequencing during the last two decades has revealed many key microbial phyla as well-represented in soil including the bacterial Proteobacteria, Bacteroidetes, Acidobacteria, Actinobacteria and the fungal Ascomycetes and Basidiomycetes. The plants can exert some pressure on the assembly of the bacterial and fungal microbiota surrounding them. Studies have demonstrated a microbial diversity gradient between the soil, the rhizosphere (the region of soil immediately surrounding the roots) and the root endosphere (inner root tissues), with less diverse microbiomes thriving inside roots compared to the rhizosphere and soil (Pascale et al., 2020; Trivedi et al., 2020). The aboveground parts of plants are also home to a diverse and variable community of microbes, deriving from the air, movement of pollinators and other insects, or other plant tissues (Vorholt, 2012). These microbes are a subset of the broader soil microbiome, and they have adapted to the unique conditions found on the aboveground parts of plants. This suggests that the soil acts as a reservoir for the microorganisms that colonize both the roots and aboveground parts of plants (Pascale et al., 2020; Trivedi et al., 2020). The soil microbiome holds great functional potential since most microbes can affect soil aggregation, can mobilize nutrients and have a role in nutrient cycling but also affect the growth and health of plants (Saleem et al., 2019; Pascale et al., 2020, Custódio et al., 2022, Liu et al., 2022). Some examples of microbial services to soil quality and plant health can be found in the section “Microbes can affect soil quality and plant health”. The importance of soil microbiota as an indicator of soil quality becomes more apparent considering that they are the source of plant-associated microbiota and can become part of our diet *via* the food web (Blum et al., 2019).

### Soil monitoring and LUCAS database

**Figure 1:**
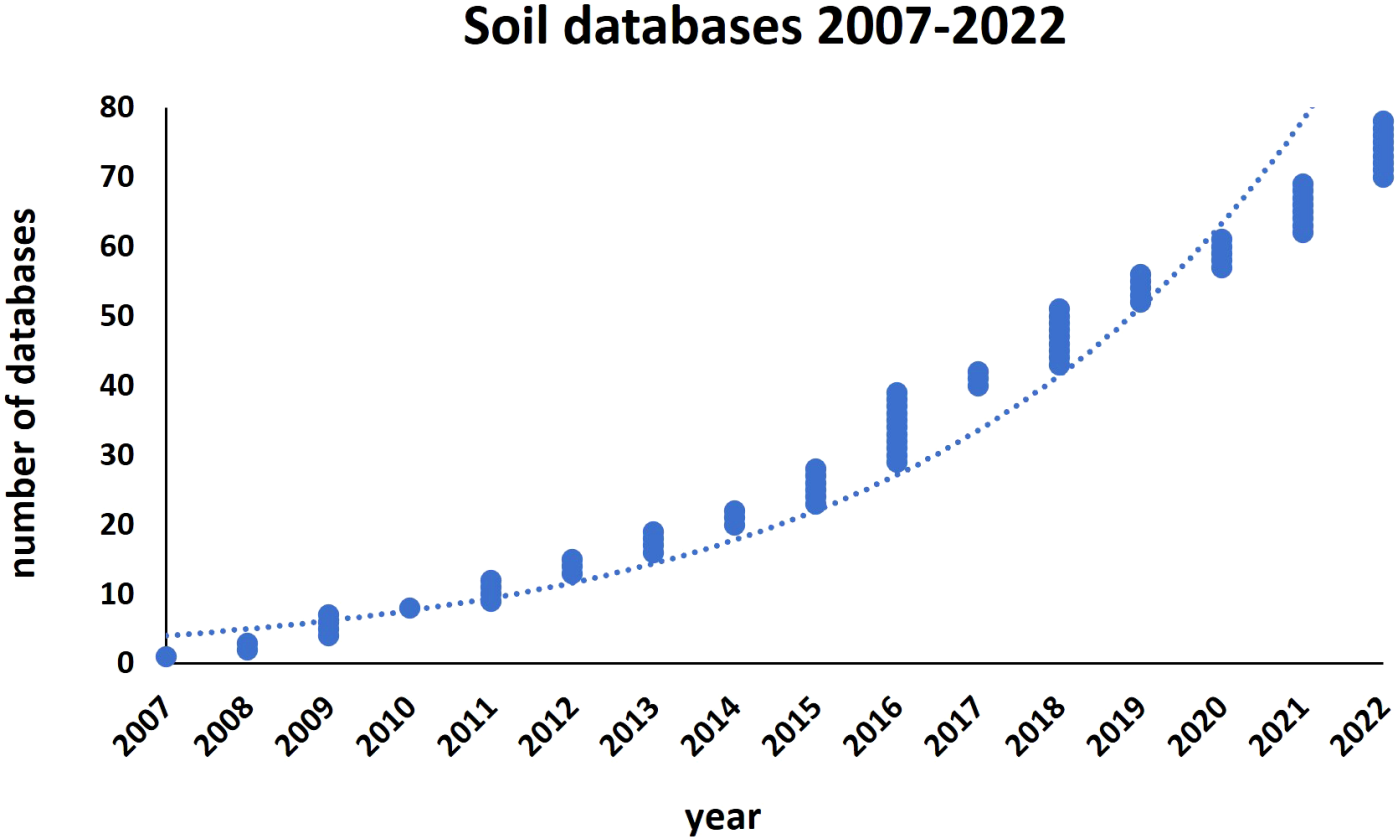
Number of European/world soil databases released from 2007 to 2022.

Due to its non-renewable nature, soil must be constantly monitored to prevent its degradation and promote long-term management. Information databases, and metastudies on soil assessment (Jian et al., 2020), facilitate the evaluation of existing and projected land production, the identification of land and water restrictions, and the assessment of risks associated with land degradation. This is critical for good management of natural resources, progress towards ending hunger, achieving food security and sustainable agriculture, especially in light of the issues caused by global climate change and the need to make the environment more resilient. In the last 15 years, the number of soil databases has rapidly increased and up-to-date there is a consistent number of those dedicated to specific soil parameters, and also those containing harmonized data from all over the world (reference https://soil-modeling.org/resources-links/data-portal/data-portal, International Soil Modeling Consortium ISMC) (**Figure 1**; **Table S1**). We present here as case-study a well-structured soil database promoted by the European Union (EU), called “Land Use/Cover Area frame statistical Survey Soil” (LUCAS) (https://esdac.jrc.ec.europa.eu/projects/lucas). LUCAS represents the largest harmonized open-access dataset of topsoil parameters available for the EU (Orgiazzi et al., 2018). The LUCAS survey, carried out by EUROSTAT on a three-yearly basis since 2006, focuses on the state and the dynamics of changes in land use and cover in the EU. Currently, all 27 EU countries have been covered and over 270,000 points have been analyzed on different land cover types (agricultural, grassland, forest, built-up areas, transport network, etc.). A standardized sampling procedure was used to collect around 0.5 kg of topsoil (0-20 cm), successfully analyzed for chemical and physical parameters like the percentage of coarse fragments, particle size distribution (% clay, silt and sand content), pH (in CaCl_2_ and H_2_O), organic C (g/kg), carbonate content (g/kg), P content (mg/kg), total N content (g/kg), extractable K content (mg/kg), cation exchange capacity (cmol(+)/kg) and multispectral properties. In 2018, the LUCAS survey included additional analyses like bulk density (*i.e*. weight of dry soil in a given soil volume), soil biodiversity, visual assessment of soil erosion and measurement of the thickness of the organic horizon in organic-rich soil. LUCAS Soil survey 2022 is currently underway. Notably, the modules Soil Biodiversity and Pesticides were recently added to LUCAS, providing tools in this direction to both policymakers and the academic community. The Soil Biodiversity module contains the largest molecular biology-based analysis of soil biodiversity in the EU. The Pesticides module provides the most comprehensive and harmonized assessment of pesticide residues in European agricultural soils (Orgiazzi et al., 2022). Acquisition of extra data will surely improve our ability to monitor soils and predict soil-mediated services. However, despite this progress, our understanding and capacity to predict the effects of soil on plants is still limited.

### The relationship between soil quality and agricultural productivity

In an agricultural set up, crop performance is dramatically influenced by the physico-chemical soil parameters, and agronomical practices are often intended to modify them to maximize field production. A fertile agricultural soil should have structure and porosity that allow the exchange of air and water, ensuring good root penetration. Improper agricultural practices can threaten soil structure *via* the breakage of aggregates, increasing soil compaction and decreasing soil porosity. This leads to a reduced soil water content and aeration, limiting root development, and crop performance in general (Rogers and Benfey, 2015).

Soil density influences root growth and shoot traits. Hard soils strongly inhibit root growth and elongation and further affect the development of leaves and shoots (Passioura, 2002). Interestingly, this inhibitory effect in hard soils has been recorded on aboveground plant traits, independently from root nutrient and water uptake, suggesting that a “soil density signal” might be sensed and systemically transmitted from roots independently from a nutritional effect (Passioura, 2002; Correa et al., 2019). In more detail, plants can adaptively respond to soil density (and in particular to soil compaction) by modifying the architecture of their root systems. This can have both positive and negative effects on plant performance, leading to either tolerance to soil compaction or a reduction in the plant yield (Correa et al., 2019). Such plastic response has a genetic component, so that “soil compaction tolerance” is to date considered a trait to be targeted in breeding programmes. On the other hand, excessively loose soils can also lead to poor crop performance, potentially due to an inadequate contact surface between roots and soil particles (Passioura, 2002). Many physical parameters influence crop productivity by affecting how the soil retains water and makes it available for crop production (Rogers and Benfey, 2015). Soil texture plays a crucial role in determining a soil’s water holding capacity and the amount of plant-available water it can provide. Different soils, such as loamy, sandy, or clay, have different abilities to attract and hold water due to the size of their pore space and the way water is held within the pores. This means that even when two soils have similar field capacity, they may not perform equally in terms of providing water for plants. In general, silt loam soils tend to provide a larger amount of plant-available water compared to other types of soil (Rogers and Benfey, 2015).

The relationship between soil nutrients and crop yield is well-established for some commercially important leafy and fruit crops (Incrocci et al., 2017). Plant nutrient availability strongly depends on their interplay with the soil physico-chemical parameters such as cation exchange capacity (CEC), water content, pH and presence of colloids. For each crop there is a range from a minimum to a maximum concentration of a nutrient that ensures the best crop performance. However, finding this range for each nutrient and for each crop is challenging. Interestingly, a meta-analysis that included different plant communities growing in different sites worldwide showed that leaf traits such as area, N and P contents were more tightly linked to nutrient availability than parameters related to plant growth (Thomas et al., 2016). This suggests that leaf traits could be used as a *proxy* to monitor nutrient availability in the soil.

As soil nutrients are taken up by roots as ions (*i.e.* ${\text{NO}}_{3}^{-}$ , ${\text{NH}}_{4}^{+}$ and ${\text{H}}_{2}{\text{PO}}_{4}^{-}$ ), the soil pH plays a pivotal role in nutrient availability. Plants can tolerate a wide range of soil pH, the optimum range for most agricultural crops is between 5.5 and 7.5, as the maximum availability of the macronutrients is displayed in slightly acidic to neutral soils. Poor crop growth and yield on acidic soils are usually due to the toxicity of H^+^, aluminum (Al), and manganese (Mn), nutrient deficiencies (particularly Ca and Mg) and reduced uptake of water (Long et al., 2017). In particular, Al toxicity is a common problem in acidic soils, where Al is present as soluble Al^3+^ ion. This form can passively enter plant roots, impairing many physiological processes and eventually impacting crop yield (Oshunsanya, 2018). Unfortunately, soil acidification is a problem that affects many cultivated areas worldwide, due to the excessive use of N fertilizers, acid rain and intensive monoculture (Long et al., 2017). Alkaline conditions affect more than 25% of the world’s soils, including calcareous, saline, and sodic soils (López-Bucio et al., 2000). High pH decreases the availability of many mineral nutrients in the soil solution causing nutrient deficiencies and reduces the soil hydraulic conductivity and soil water retention (Suarez, 2012; Xu et al., 2020). Beside the negative effect on nutrient uptake, salinity and sodicity mainly impact plant growth through ion toxicity and osmotic stress (Huang et al., 2017), while high pH affects root cell elongation and root water transport (Xu et al., 2020). All these mechanisms eventually lead to reduced crop growth and productivity as demonstrated for rice, where grain yield was shown to significantly decrease with increasing pH in a range from 7 to 9 (Huang et al., 2017).

While the relationship between individual soil parameters, such as nutrient content, soil structure, or pH, and crop performance is clear in principle, it becomes more complicated and unpredictable in field conditions. This is because multiple physico-chemical soil parameters can affect soil quality simultaneously, creating a unique environment for each field. This indicates that methods that consider multiple parameters can be more accurate to predict their effect on plant performance than single-use parameters. As an example, Peralta et al. (2014) studied the relationship between different soil parameters and wheat nitrogen demand and grain yield in different sites. Despite a positive correlation between N availability and grain yield, the study also showed that the parameters more relevant to explain the wheat N demand were mostly site-specific. This highlights the importance of considering the “site” variable when analyzing soil factors and their impact on crop performance. In a study focusing on wheat yield in hilly regions they found that spatial variability significantly affected crop performance, underscoring the importance of considering the “site” variable when analyzing soil factors and their impact on crop performance. The study also showed that topographic attributes, such as slope, can have a dramatic impact on water availability and should be integrated into crop performance predictions (Ajami et al., 2020). Furthermore, the annual weather conditions could influence the physico-chemical soil features that are critical for crop yield. That was suggested by Juhos et al. (2015) that performed a multivariate analysis on the same fields using data coming from 10 years and considering different crops (maize, wheat, sunflower). They found that in droughty years, the salinization, soil texture, and nutrient contents were the most influencing parameters, while in rainy years the SOM and the nutrient contents were the main limiting factors determining the crop yields.

### Microbes can affect soil quality and plant health

Microbiota and soil quality have a reciprocal relationship, as evidenced by various examples in the literature. Microbes can directly affect soil quality through their ability to release extracellular polymeric substances (EPS), which are mostly composed of polysaccharides, proteins, and DNA. These substances improve soil particle aggregation and help retain moisture (Costa et al., 2018). In addition, arbuscular mycorrhizal fungi (AMF) can form dense networks of hyphae that facilitate nutrient transfer between different soil areas and also contribute to maintaining soil structure and aggregation (Rillig et al., 2015). In the next examples, we will use plant health as a readout of soil quality. Although the term “plant health” can be interpreted in multiple ways, we aim to define a plant as healthy “as long as its physiological performance, determined by its genetic potential and environmental conditions, is maintained” (Döring et al., 2011 and references therein). An ideal soil for agricultural production should accommodate a diverse microbiome containing many microbes with enhanced nutrient mobilization capacity and the ability to suppress plant pathogens (Weller et al., 2002; Saleem et al., 2019). Many studies have shown that when plants are stressed, they “cry for help” by emitting chemical signals that attract helpful soil microbes that can assist in relieving the stress (Pascale et al., 2020). For instance, when soil P or N is unavailable for plants, they secrete molecules to attract AMF and nitrogen-fixing rhizobia from the soil that can facilitate P and N uptake respectively (Oldroyd, 2013; Lanfranco et al., 2018). When plants experience drought, they can also recruit microbes that can specifically attenuate drought-related stresses in plants (Williams and de Vries, 2020). Similarly, in soils with unavailable iron (Fe) plants release metabolites to attract iron-mobilizing microbiota (Stringlis et al., 2018; Harbort et al., 2020). In the case of increased pathogen load in soil and a disease outbreak, there is an enrichment of selected microbiota *e.g.* fluorescent *Pseudomonas* sp. that can lead to the suppression of disease (Weller et al., 2002). This phenomenon is known as specific soil suppressiveness to plant diseases. However, disease suppression can be also achieved by increasing the activity of soil microbiota *via* soil manipulation and the addition of organic matter, certain agronomic practices, or improvement of soil fertility, leading to general suppressiveness (Raaijmakers and Mazzola, 2016). Additionally, the presence or absence of rare bacterial taxa in the soil can determine whether the soil can favor future disease outcomes, opening new avenues toward the use of microbes as indicators of “disease-prone” soils (Wei et al., 2019). Finally, drought can disrupt soil structure and water content and in turn affect the structure and activity of microbiomes. Severe drought can affect the presence of microbiota that can contribute to plant resilience (Santos-Medellín et al., 2021), while conditions of mild drought can increase bacterial activity related to Fe transport and metabolism (Xu et al., 2021). Therefore, achieving plant tolerance to drought requires decoding how soil and plant microbiomes respond and adapt to drought conditions. Caution is needed under conditions of (a)biotic stress, since plants may have less control over their microbiota, leading to the formation of dysbiotic plant microbiomes. Dysbiosis can affect the host, the microbiota, or both, and occurs when the proportions of microorganisms in the microbiome are not balanced or when harmful microorganisms are present in high numbers. This can lead to negative effects on the plant, such as reduced growth and increased susceptibility to disease. It is important to take steps to prevent or mitigate dysbiosis in plant microbiomes to maintain the health and productivity of the plants (Arnault et al., 2022).

### Monitoring soil quality in agriculture through soil-plant-microbiota crosstalk

The expansion of ecosystem services provided by soils and their connection with human, animal and ecosystem health underpins the key role of soil in the emerging “*One Health*” vision (Banerjee and van der Heijden, 2022; Lehmann et al., 2020). The numerous health indicators for soil (physical, chemical and biological) and the complex interactions between them in many different processes makes it challenging for soil scientists to select the most representative indicators or to determine how many indicators should be considered during soil health evaluation (Creamer et al., 2022). Moreover, in different climatic conditions the same indicator could be considered high or low depending on other parameters (for example organic C levels can be considered high or low depending on the soil clay content).

Recently, the EU soil mission launched a new set of eight objectives proposing clear targets and new measurable physical, chemical, biological and socio-economic indicators for monitoring the state of the soil (European Mission - A soil deal for Europe). However, not all of the proposed parameters can be used to assess different ecosystems, and the range of indicators is somewhat limited and may not be fully representative of specific contexts. In general, biological properties are under-represented in soil health evaluations, despite their direct link to physico-chemical parameters and their recognized impacts on soil fertility, plant productivity, and human health (Bongiorno, 2020). For instance, while the EU has recognized the importance of improving soil biota quality as part of its trajectory toward sustainable soil management and restoration, the monitoring of microbiota dynamics and biodiversity is not included as a critical soil indicator. Novel criteria and indicators based on the tracking of microbiota diversity should be introduced to evaluate soil quality. This is feasible considering the advances in next-generation sequencing technologies that have been extensively used in the last two decades toward microbiota mapping in different ecosystems, including soils. However, some challenges still exist before adopting soil microbiota-based indicators: a) while taxonomy and composition can be easily tracked, the functionality of microbiomes relies on more expensive and bioinformatically demanding approaches such as shotgun metagenomics and metatranscriptomics, and b) the existing microbial databases, usually used for microbiome characterization and functionality, might not be representative of most soils and ecosystems. In this framework, the use of microbiota diversity as a soil quality indicator is not yet widely adopted. Given that microbial colonization alters plant physiology, development and gene expression, it may be possible to monitor host plant traits and use plant health as a reliable soil quality indicator.

**Figure 2:**
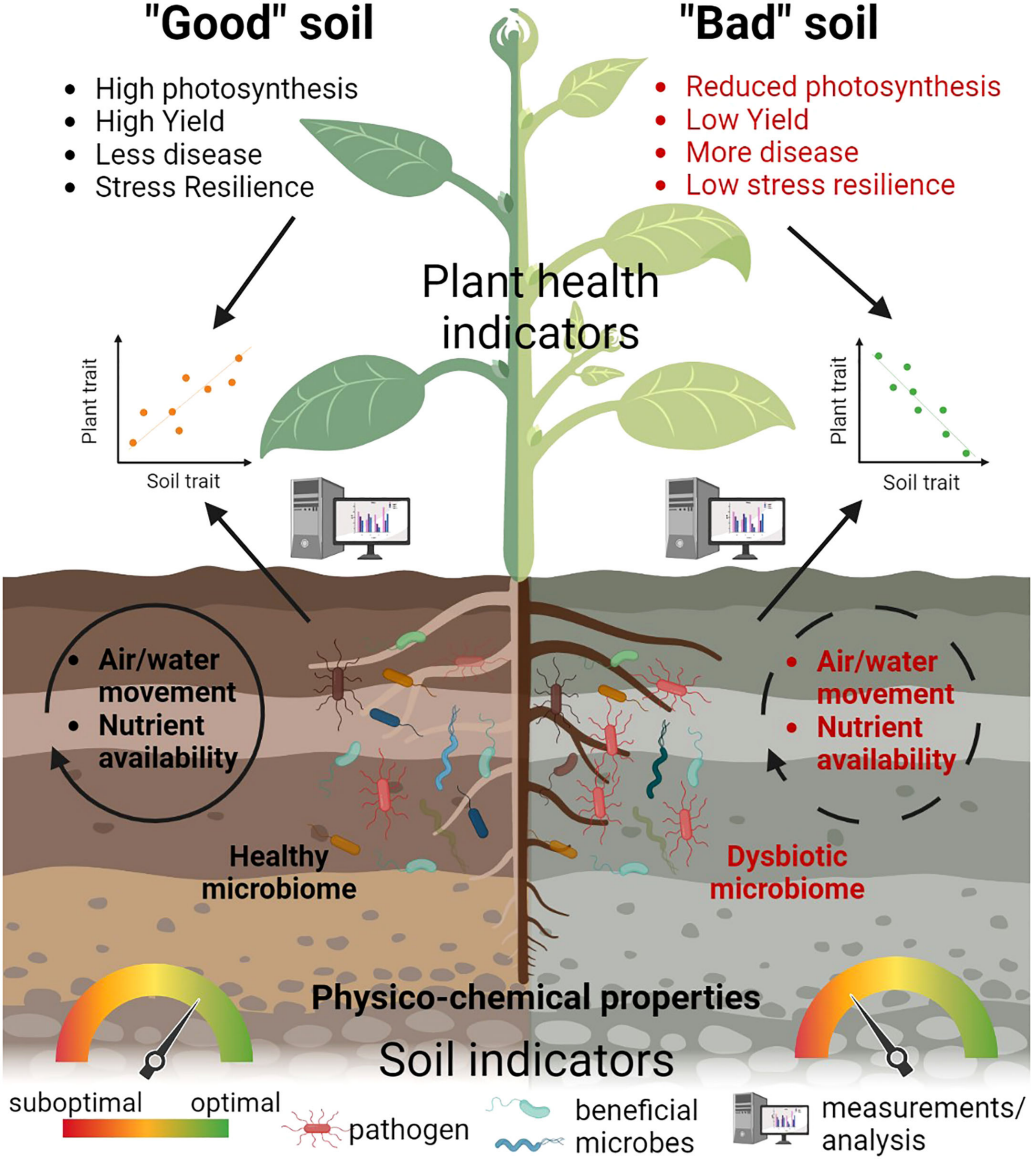
Plant health indicators as a *proxy* of soil quality. A combination of plant health parameters such as photosynthesis, yield and resilience can represent a powerful ‘‘lens’’ to evaluate soil quality and differentiate between a “good”, healthy soil and a “bad” soil, with properties not supporting plant growth. In a “good” soil, a diverse and balanced microbiome is present, along with optimal physico-chemical parameters and air and water movement, while different nutrients are available for the plant and support its growth, health and resilience. In contrast, a “bad” soil is characterized by a dysbiotic microbiome, reduced water and air movement, limited nutrient availability and suboptimal physico-chemical parameters, which negatively affect various plant traits. Using different methods and analyzing multiple indicators, we can determine the relationship between soil parameters and selected traits of plant health.

Indeed, plant health can be considered as the final output of the integration of many soil quality parameters since it reflects the complexity of the interaction between plants and their environment into a single outcome. Plants can therefore be seen as a powerful lens through which we can interpret the soil, as already suggested by ecologists (van der Putten et al., 2013). This approach can offer a real-time and up-to-date methodology to interpret the status of a plant by focusing on multiple reliable measurements, rather than just plant yield (**Figure 2**). Each of the suggested plant health parameters represents an important *proxy* for different soil quality parameters, including soil microbial diversity. By using plant health as an indicator of soil quality, we could monitor and address important societal goals, such as reducing soil pollution, improving soil restoration and enhancing soil structure to support habitat quality and soil biota and crops.

As an example, we are here listing a set of plant health parameters that are easy to measure and essential to evaluate soil status (**Table 2**). The plant photosynthetic capacity likely mirrors important information about soil chemical parameters, such as nutrient availability (Ai et al., 2017), but it is also affected by soil physicochemical and biological parameters, such as root-associated microbes (Moustakas et al., 2020; Balestrini et al., 2020) or conditions affecting these parameters like desertification (Qiu et al., 2018), pH (Long et al., 2017). It is a sensitive indicator, with rapid and large changes influenced by external factors. Chlorophylls, including Chl a, Chl b and Chl a+b, can be easily measured with a simple ethanol-based extraction and colorimetric assays or with on-field portable approaches. A correct range of photosynthetic pigment concentration correlates with a healthy content of macro- and micro- nutrients. Another plant parameter useful to infer crucial soil physico-chemical indicators and microbiota dynamics is represented by the plant elemental composition (Callaham and Stanturf, 2021; Schjoerring et al., 2019; Shaaban et al., 2016; Mohiuddin et al., 2022; Singh et al., 2022) (**Table 2**). The macro- and micro-elements accumulating in plant tissues provide a clear readout of soil nutrient availability that can impact plant food security and human nutrition, therefore mirroring a meaningful soil service. In addition, detailed nutritional profiling of plant tissues can facilitate precision in fertilizer management and can be performed at different stages of plant growth. Monitoring it over time can provide a better resolution and improve our ability to interpret soil nutrient availability and its fluctuations. Furthermore, tracking the presence of pollutants in plant tissues can also provide insights into soil health, including its levels of heavy metals, herbicides, pesticides and microplastics, which are all absorbed by plant roots and accumulate in plant tissues (Biswas et al., 2018; Stoykova and Inui, 2021; Walder et al., 2022; Edlinger et al., 2022). Altogether, monitoring the levels of different target molecules accumulating inside plant tissues could help us fingerprint the quality of soils.

**Table 2:** Plant health indicators that correlate with soil characteristics.

	Soil parameters		
Plant health indicator	Physical	Chemical	Biological
**Photosynthetic capacity**	Desertification (Qiu et al., 2018) Water (Qiu et al., 2018)	P and N soil availability (Ai et al., 2017)	AM fungi (Moustakas et al., 2020; Balestrini et al., 2020)
**Micro- and macro- elements**	Soil texture (Callaham and Stanturf, 2021)	NPK and SOM (Schjoerring et al., 2019); pH, redox potentials (Mohiuddin et al., 2022); electric conductivity (Shaaban et al., 2016)	Microbiota diversity (Singh et al., 2022); AM fungi (Lanfranco et al., 2018)
**Pathogenesis**	Structural heterogeneity and water/oxygen availability (Otten and Gilligan 2006); moisture and clay content (Döring et al., 2020)	NPK, organic matter content and cation exchange capacity (van Agtmaal et al., 2018)	AM fungi (Vannini et al., 2021); microbiota (Chialva et al., 2018; Chialva et al., 2022)
**Stress resilience**	Water repellency, soil texture and macropore density (Seaton et al., 2020)	Soil carbon stock and pH Seaton et al., 2020); soil indigenous nutrients (Deng et al., 2020)	Microbiota (Seaton et al., 2020; Trivedi et al., 2022)
**Presence of pollutants (heavy metals, herbicide, pesticide, microplastics)**	Temperature and moisture (Biswas et al., 2018)	Organic matter and mineral fractions (Biswas et al., 2018)	Microbial activities (Biswas et al., 2018); microbiota (Walder et al., 2022); AM fungi (Edlinger et al., 2022)

Soil and plant resilience are considered two sides of the same coin. Seaton et al. (2020) and Deng et al. (2020) showed that several physico-chemical soil parameters such as water repellency, texture, pH, SOC and nutrient soil content can affect the ability of plants to withstand (a)biotic stresses. Additionally, alterations in the composition and activities of the plant-associated microbiota can affect plant resilience to biotic and abiotic stresses (Chialva et al., 2018; Pascale et al., 2020; Jansson and Hofmockel, 2020; Chialva et al., 2022; Vannini et al., 2021; Trivedi et al., 2022). It is also suggested that plant adaptation to climate change will be driven by eco-evolutionary interactions with its associated microbiome (Trivedi et al., 2022). Metatranscriptomic and metagenomics technologies could be used to identify plant-associated microorganisms and their relationship to soil characteristics and plant adaptation. Moreover, meta-omics studies provide information about gene expression levels *via* genomics and transcriptomics, as well as about post-translational changes *via* proteomics. Additionally, metabolomics or volatilomics can provide information about metabolites as a result of cellular processes, or volatile organic compounds (VOCs), respectively. Therefore, a combination of -omics methods can connect all aspects of the plant-microbe-soil relationship at the cellular level (Sharma et al., 2020; Brown et al., 2021; Gupta et al., 2021; Mishra et al., 2022; Yu et al., 2022).

## Future perspectives

In this review, we argue for the use of plant health traits as indicators of soil quality, under both a physico-chemical and a biological point of view. However, their adoption needs to be accompanied by the development of rapid and reliable assays on both the plant and soil sides. On the plant side, new strategies suitable for tracking plant health may utilize gene-expression based technologies or bioinspired soft and miniature machines able to record a wide range of parameters. Since it has already been demonstrated that RNAs and miRNAs can be used as marker genes to monitor the onset and transmission of (a)biotic stresses (López-Galiano et al., 2019; Tyagi et al., 2021; Šečić et al., 2021; Zhang et al., 2022; Paes de Melo et al., 2022) species-specific large-scale microarrays which cluster all the (a)biotic stress markers genes available in shoot could be envisaged. Additionally, plant-like miniature adhesive systems for *in situ* leaf microenvironment monitoring have been recently developed (Fiorello et al., 2021; Coatsworth et al., 2022). The development of plant-inspired miniature machines coupled by sensors to detect in real-time macro- and micronutrients in plant leaves would be instrumental to infer soil nutrient availability and open avenues for their application in precision agrotechnology systems. Additionally, the agriculture 4.0 technologies applied in the field such as the use of drones and sensors could provide novel ways to assess plant health. Drones can serve as a powerful tool to monitor the growth of crops (Rejeb et al., 2022). For example, new spectroscopic techniques, such as unmanned aerial vehicle (UAV) multispectral sensor is already used in the field for monitoring crop leaf N accumulation, leaf area index, and leaf dry weight and photosynthetic capacity (Potgieter et al., 2017; Yao et al., 2019; Zhang et al., 2020; Sharma et al., 2022). This drone-based phenotyping approach might be further fine-tuned and fostered to develop a non-destructive, rapid and *in situ* method to track plant health. On the soil side, applying methods to detect the actual level of microbial biodiversity, as well as microbiota functions relevant to plant growth promotion and the regulation of biogeochemical cycling would be a major step forward. Up-to-date techniques for genome/amplicon sequencing and subsequent bioinformatic analyses are already available, and the costs and time from sequencing to final data are progressively reducing. That could make this strategy potentially more affordable in the future for daily practice in the field. Together, the systems- and molecular-level knowledge on plant-soil-microbiota crosstalk will be pivotal to decode and improve soil health and crop productivity.

## Author contributions

VF, SP, IAS proposed the concept. MG, AS and VF organized and drafted the manuscript. SP, IAS contributed to the editing of the manuscript. VF, SP and IAS supervised the work. All authors have read and approved the manuscript.
